# Host-Plant Selectivity of Rhizobacteria in a Crop/Weed Model System

**DOI:** 10.1371/journal.pone.0000846

**Published:** 2007-09-05

**Authors:** Simon L. Zeller, Helmut Brandl, Bernhard Schmid

**Affiliations:** Institute of Environmental Sciences, University of Zürich, Zürich, Switzerland; University of California at Davis, United States of America

## Abstract

Belowground microorganisms are known to influence plants' performance by altering the soil environment. Plant pathogens such as cyanide-producing strains of the rhizobacterium *Pseudomonas* may show strong host-plant selectivity. We analyzed interactions between different host plants and *Pseudomonas* strains and tested if these can be linked to the cyanide sensitivity of host plants, the cyanide production of bacterial strains or the plant identity from which strains had been isolated. Eight strains (four cyanide producing) were isolated from roots of four weed species and then re-inoculated on the four weed and two additional crop species. Bacterial strain composition varied strongly among the four weed species. Although all six plant species showed different reductions in root growth when cyanide was artificially applied to seedlings, they were generally not negatively affected by inoculation with cyanide-producing bacterial strains. We found a highly significant plant species x bacterial strain interaction. Partitioning this interaction into contrasts showed that it was entirely due to a strongly negative effect of a bacterial strain *(Pseudomonas kilonensis*/*brassicacearum*, isolated from *Galium mollugo*) on *Echinochloa crus-galli*. This exotic weed may not have become adapted to the bacterial strain isolated from a native weed. Our findings suggest that host-specific rhizobacteria hold some promise as biological weed-control agents.

## Introduction

The growth of a plant is influenced by the soil environment surrounding the roots. Feedback effects between soil microorganisms and plants affect not only individual plant species positively or negatively but can reduce or enhance local plant diversity [1]. Such interactions take place in the rhizosphere, the zone of the soil which is directly influenced by the plant's roots. Rhizosphere-inhabiting bacteria, also termed rhizobacteria, are adapted to colonize and compete for space on and around plant roots [2]. Rhizobacteria can influence plants in various ways, depending on their ability to excrete metabolites, incorporate root exudates or compete with other soil microorganisms.

The group known as deleterious rhizobacteria (DRB), which are defined as non-parasitic plant pathogens [3], have been shown to variously excrete cyanide, phytohormones and phytotoxins that can affect the metabolism of plants negatively [4]. It has been suggested that cyanide is the major inhibitory compound that leads to significant growth reduction in some plant species such as *Lactuca sativa* and *Echinochloa crus-galli* [5].

There are indications that some rhizobacterial strains show strong host-plant selectivity and colonize a single plant species or variety. For example, a particular *Pseudomonas* isolate caused a large reduction in the growth of some *Pisum sativum* cultivars but had no effect on other cultivars or on wheat [6]. Host-plant selectivity might also be involved in a long known agricultural phenomenon. Schippers *et al.* [7] showed that yields of well-fertilized potato fields decrease when the same fields were farmed continuously for several years. They found abundant cyanide-producing pseudomonads on potato roots and concluded that accumulation of such microbes may cause the negative effects. It is conceivable that continuous farming may lead to an enrichment of host-plant selective DRB which would then inhibit plant growth.

In a previous experiment involving 24 grassland species (Petermann *et al*., in review) we found a strong advantage of “away” over “home” plant species [8] in competition on soils obtained from monocultures of the latter. In this context, “home” means that plants were grown on soil collected from 3-year old monocultures of the same species and “away” means that plants were grown on soil collected form 3-year old monocultures of a different species. Four of the 24 species studied, including the one with the most negative home effect, *Echinochloa crus-galli,* were used in the present study. Here we used this terminology to describe host plant–bacterial strain interactions. The term “home” refers to an inoculation of a plant with a bacterial strain isolated from that plant species and “away” when a bacterial strain was used to inoculate a different plant species. Our aim is not only to deliberately search for interactions but ultimately to investigate their function in the ecosystem.

Microbial communities of four weed species were assessed, with the expectation of finding varying bacterial compositions among plant species, thereby focusing on the occurrence of cyanide-producing *Pseudomonas* strains. The ability of four weed and two crop plant species to tolerate cyanide was then assessed in a bioassay using seedlings. A full factorial experiment allowed us to identify interactions between eight isolated rhizobacterial strains (four of them cyanide-producing) and the six plant species. Monocultures of plants and bacterial strains were used to investigate these interactions. In this experiment, we expected to find that cyanide-resistant plants would not be affected by cyanide-producing rhizobacteria. Furthermore, we hypothesized that bacterial strains might be better able to colonize their “home” than an “away” plant species and that this would be detectable if cyanide-producing DRB isolated from the rhizosphere of one plant species would inhibit the same plant species more than others.

Rhizobacteria that reduce the growth of weed plants but do not affect crops could potentially be used as biological herbicides [5]. A better understanding of such plant-bacteria interactions is required to develop such novel agricultural applications.

## Materials and Methods

### Plant species and isolation of rhizobacteria

Root samples of the four herbaceous species *Echinochola crus-galli* L., *Hordeum murinum* L., *Centaurea jacea* L. and *Galium mollugo* L.were collected. These species commonly occur in weedy plant communities or have weedy or invasive relatives. Here we treated them as model weeds and therefore call them weeds in the following. The plants grew for 3 years in experimental monocultures on sandy-loamy soil (pH = 7.6±0.2, soluble nitrogen = 26±0.9 mg kg^−1^, soluble phosphorus = 4±0.3 mg kg^−1^) at the agricultural extension station Forschungsanstalt Agroscope Reckenholz-Tänikon ART in Zurich, Switzerland. Soil samples containing roots were obtained from three plants per species. They were bagged immediately to prevent contamination and stored at –80°C until processing. After 2–3 months, the soil samples were immersed into sterile 0.9% NaCl solution and shaken to separate the roots form the soil. Root pieces were exposed to an ultrasound source (10 min, 286 W; Transsonic T 460/H, Elma®, Singen, Germany) to remove the bacteria attached to the root surface. Rhizobacteria were cultured on King's B agar plates [9]. This medium is selective for *Pseudomonas* species but also allows other bacteria to grow [10]. We were therefore able to acquire and compare a relatively diverse set of rhizobacterial strains. Estimates of the bacterial Colony-Forming Units (CFU) per g of root were made by the Most Probable Number (MPN) method [11] after 48 h of incubation at 28°C. Representative single colonies with distinct morphological traits (pigment, colony form, fluorescence and opacity) were selected from each plate and streaked onto non-selective nutrient agar (NA) [12]. The aim was to acquire a maximally diverse set of microbes from the rhizospheres of each of the four weed species. All isolates were sub-cultured at least twice and examined microscopically to obtain pure cultures (e.g. no fungal contamination). The whole isolation process was repeated until 65 strains were isolated. Cultures were stored in slanted NA tubes at 4°C and in Eppendorf® tubes at –80°C.

In addition to the four weed species, two crop species, *Triticum aestivum* L. cv. ARINA (supplied by the Institute of Botany, University of Zurich, Switzerland) and *Daucus carota* L. cv. NANTAISER II (purchased from Coop AG, Basel, Switzerland) were used as counterpart to the four weed species. No rhizobacteria were isolated from these plants. Of the six model plants in total, three were monocotyledonous *(E. crus-galli, H. murinum, T. aestivum)* and three were dicotyledonous *(C. jacea, G. mollugo, D. carota)*.

### Characterization and identification of bacterial strains

All strains were characterized using a phase-contrast microscope and standard microbiological tests (Gram staining, Oxidase test). To facilitate the identification, only Gram-negative bacteria were used in the further experiments. Cyanide tests were performed with 49 gram-negative strains in the following way: 0.2 g glycine was added to NA to enhance cyanide production. Strips of filter paper were immersed in Tetrabase solution [13] and attached to the lids of Petri dishes using parafilm®. After 24–48 h, a color change from white to deep blue indicated the production of HCN inside the sealed Petri dish. The cyanide test was repeated once. *Pseudomonas fluorescens* strain CHA0, from our strain collection, was used as positive control.

Analytical Profile Index tests (API 20NE, bioMérieux® SA, Lyon, France) were used to identify 31 gram-negative, oxidase-positive isolates. These tests combine 8 biochemical and 12 substrate growth tests on a single slide. They results in a colour code that is entered into a database. It was often not possible to identify the strains to species level. In many cases, two or three possible species names were given. However, we were able to differentiate between more similar and more different strains. The identification procedure of 12 strains was repeated once to assess the reliability of the API 20NE system. In 6 cases, one of the 20 tested parameters changed but this did usually not change the final identification. After the completion of all experiments (see below) one particularly interesting isolate was identified by DSMZ (German Collection of Microorganisms and Cell Cultures, Braunschweig, Germany).

Two isolated, putative *Pseudomonas fluorescens* strains of each of the four weed species were used in host-selectivity experiments. Since we were interested in deleterious bacteria, we chose cyanide-producing strains if possible. If no such strains were available, other fluorescent pseudomonads (E24, E 211, H32 and H310) were used.

### Cyanide sensitivity experiment for the six test-plant species

A previous study reported that different plant species can react differentially to cyanide exposure [5]. It is not known whether cyanide tolerance is common in weeds. Since some of the isolated bacteria produced cyanide, it was important to determine the effect of cyanide on our six model plants. The ability to tolerate cyanide was assessed with a modified root-reduction bioassay [5]. All seeds were surface-sterilized by immersing them in 6% NaOCl solution for 3 minutes to reduce attached microbes and then rinsed thoroughly with sterile water. Germination took place in Petri dishes with sterile filter paper in a climate chamber (25°C, 16/8 h light/dark). To reduce fungal growth, 1–2 ml 0.1% of a commercially available fungicide (Carbendazim® FL SA 60, 0.1%, SINTAGRO AG, Härkingen, Switzerland) was added. We were therefore able to keep fungal contaminations under control. For each plant species, 1–4 seedlings with a short but well-developed root were placed on water agar plates containing concentrations of 0, 2, 5 and 10 mg KCN/l. All cyanide-agar mixtures were adjusted to pH 7 by adding NaOH. Pilot experiments performed with *D. carota* plants showed that such cyanide doses reduce the root growth (2 mg/l, 5 mg/l) or stop it entirely (10 mg/l; data not shown). The plates were then incubated in a randomized array in a dark climate chamber at 25°C. After two days, the number of roots and the length of the longest root were recorded. The experiment was repeated four times.

Due to technical difficulties, only few studies tried to quantify cyanide levels directly in the soil. One report shows, that rhizobacteria release on average 2.5 mg KCN l^−1 ^during a period of 36 h [14]. However, under optimal conditions, *P. fluorescens* strains may produce up to 110.7 mg KCN l^−1^ [15]. Natural soils may also contain traces of cyanide (0.085 µg g^−1^; [16]) but only in much lower concentrations. To guarantee ecological relevance of this experiment, we choose a range of KCN concentrations that lies well above the soil background and covers a wide margin around the average cyanide production rate of rhizobacteria.

### Host-plant selectivity experiment

This experiment was designed to investigate the influence of the eight isolated bacterial strains on the four weed and two crop species. Each of the 48 bacterial strains x plant species combinations was replicated four times. Bacterial solutions were prepared the day before the start of the experiment. Two-day old cultures in NA were centrifuged at 4400 g for 10 min. The pellet was then re-suspended in 0.05M MgSO_4_ and adjusted to an optical density at 600 nm (OD600) of 0.82–0.87. A counting chamber was used to translate the OD600 values into numbers of CFU's. Bacterial solutions were kept at 4°C or on ice until inoculation.

One replicate of a treatment combination consisted of four surface-sterilized and pre-germinated seedlings planted in a 9.5×9.5 cm square pot containing pasteurized (2×24 h at 80°C) BF4 perlite potting soil (Tref, GVZ-Bolltec AG, Zürich, Switzerland; moisture content = 70%; pH = 5.8; organic matter of dry substrate = 50%, C = 30%, N = 0.05%). Immediately after planting, each seedling within a pot was inoculated with 0.5 ml 0.05M MgSO_4_ solution containing 4.0–4.3×10^8^ CFU bacteria. For each plant species, we prepared four additional pots with four seedlings each. These served as controls and received sterile MgSO_4_ solution. After inoculation, a thin layer of sterile plastic granulate (Elastollan® C95A10, BASF, Wyandotte, USA) was added to cover the soil around the seedlings and thus reduce airborne contamination with microorganisms and evaporation [17].

The inoculated pots were then placed in a completely randomized array in a climate-controlled glasshouse (20–25°C, 12 h light/dark). The pots were kept sufficiently moist by daily watering and we re-randomized their position weekly. Shoot length and number of leaves were recorded every fortnight. After six weeks, the aboveground plant parts were harvested, dried and weighed. Root samples were taken from five selected pots to assess the bacterial populations after the harvest was finished.

### Data analysis

Data from the cyanide screening and glasshouse experiments were analysed separately using general linear models and analysis of variance (ANOVA). For the host-plant selectivity experiment we used a factorial ANOVA with plant species and bacterial strains as main effects. The interaction was decomposed into several contrasts that are typically made in interaction studies such as reciprocal transplant experiments [8]. The most important contrasts that we tested are described in the Results section. Significance level was α = 0.05 in all analyses. Transplanted seedlings of the host-plant selectivity experiment which started to grow with a delay of two or more weeks were excluded from the analysis (12 of the 864 seedlings). Residual plots were examined to identify outliers and check if the assumptions of normality and homoscedasticity were valid.

## Results

### Identification and characterisation of the rhizobacterial community

We isolated 65 rhizobacterial strains with *Pseudomonas*-selective King's B agar. As described by Kremer *et al.* [9] not only pseudomonads but also Gram-positive and spore-forming bacteria are able to grow on this medium. Thirty-one fluorescent and gram-negative strains were selected for identification with API 20 NE tests (Table 1). We could distinguish 25 different bacterial strains. In four cases, more than one strain showed a similar API test result. These strains were treated as identical. Each weed species appears to have a plant-specific rhizobacterial community. Only two bacterial strains colonised the rhizospheres of more than one species, namely the two grass species *E. crus-galli* and *H. murinum*.

**Table 1:** Rhizobacterial communities of four weedy plant species.

Strain ID	CN^−^	Bacterial species a	Gal	Cen	Ech	Hor
G11^b^,G25	Yes	*Pseudomonas kilonensis/brassicacearum* c	X			
G21,G22		*Pseudomonas fluorescens, Burkholderia cepacia* A^d^	X			
G23		*Pseudomonas fluorescens, Pseudomonas orryzihabitans*	X			
G27^b^	Yes	*Pseudomonas fluorescens* A	X			
C22		*Ralstonia picketti, Pseudomonas fluorescens*		X		
C25		*Ralstonia pickettii*		X		
C27^b^	Yes	*Pseudomonas fluorescens/aeruginosa, Ralstonia pickettii*		X		
C210^b^	Yes	*Pseudomonas fluorescens* B		X		
E11		*Chryseobacterium indologenes*			X	
E12		*Aeromonas hydrophylia/caviea*			X	
E21		*Pseudomonas fluorescens*, *Burkholderia gladioli*			X	
E22		*Sphingomonas paucimobilis*			X	
E26		*Pseudomonas putida* A			X	
E211^b^		*Pseudomonas fluorescens* C			X	
E25,H12,H13		*Rhizobium radiobacter*			X	X
E24^b^,E212,H36		*Pseudomonas fluorescens, Burkolderia cepacia* B			X	X
H21		*Pseudomonas luteola*				X
H22		*Weeksella virosa, Empedobacter brevis Brevundimonas vesicularis*				X
H23		*Comamonas testosteroni, Pseudomonas alcaligenes*				X
H24		*Aeromonas hydrophyla/cavia, Pseudomonas luteola*				X
H32^b^		*Pseudomonas fluorescens, Burkolderia cepacia* C				X
H33		*Delftia acidovorans*				X
H34		*Stenotrophomonas maltophilia*				X
H310^b^		*Pseudomonas fluorescens, Ralstonia pickettii Burkholderia cepacia*				X
H312		*Pseudomonas putida* B				X

Selected rhizobacterial communities of the four weedy plant species *Galium mollugo* (Gal), *Centaurea jacea* (Cen), *Echinochloa crus-galli* (Ech) and *Hordeum murinum* (Hor). Each row represents a different bacterial strain. The ability to release cyanide (CN^-^) is indicated by Yes.

a Species name given by API 20NE® identification system. If no clear identification was possible, the two or three most likely bacterial species names were recorded.

b Strains used in the host-plant selectivity experiment.

c Strain identified by DSMZ.

d A-C: Strains differing in biochemical tests but identified as similar species.

Qualitative cyanide tests were performed with the 31 API tested isolates. Five *Pseudomonas* strains (G11, G25, G27, C210 and C27) isolated from the two dicotyledonous species *G. mollugo* and *C. jacea* produced cyanide in measurable concentrations. The strains C27 and G27 showed a faster blue coloration of the test strips than the other three strains. However, this could be due to faster growth of these microorganisms on the given media (data not shown). DSMZ (Braunschweig, Germany) assigned strain G11 to be either *Pseudomonas kilonensis* [18] or *Pseudomonas brassicacearum* [19] with a similarity of 100% based on morphology, motility, utilization of carbon sources, cellular fatty acid composition and partial 16S rRNA sequencing. These very closely related species are difficult to distinguish with the methods used. The identification based on the 16S data deviates from the API 20 NE analysis, where the strain was identified as the closely related *P. fluorescens*. This suggests that other identifications based solely on the API 20 NE tests may be inaccurate *(P. kilonensis/brassicacearum* was discovered only recently and is therefore not yet part of the API 20 NE strain library). Despite this, the identifications given in Table 1 are at least valid “morphospecies” fulfilling the requirements for our home vs. away test of host-plant responses to colonizing bacterial strains.

### Cyanide-sensitivity bioassay

**Figure 1:**
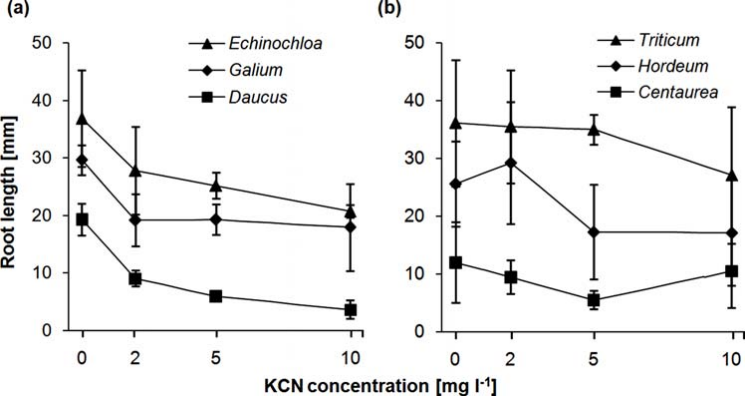
Cyanide-sensitivity bioassay. Reduction in root length caused by cyanide (KCN) application to weed and crop seedlings: (a) group of plant species that showed a more sensitive reaction to cyanide, (b) group of plant species that showed a less sensitive reaction. The error bars indicate the standard deviation of the means (n = 4). Only the names of the plant genera are given (for species names see text).

Cyanide had a negative influence on the root growth of all tested plant seedlings (F_1, 3906_ = 71.899, P = 0.0001). However, the reaction to cyanide varied widely between the six plant species. *Echinochloa crus-galli*, *G. mollugo* and *D. carota* showed significantly stronger root-growth inhibition than *T. aestivum*, *H. murinum* and *C. jacea* in response to cyanide exposure (interaction cyanide concentration x contrast *‘E. crus-galli*/*G. mollugo*/*D. carota* versus *T. aestivum*/*H. murinum*/*C. jacea’*: F_1, 332.7_ = 6.1233; P = 0.0482; Fig. 1).

### Host-plant selectivity experiment

**Figure 2:**
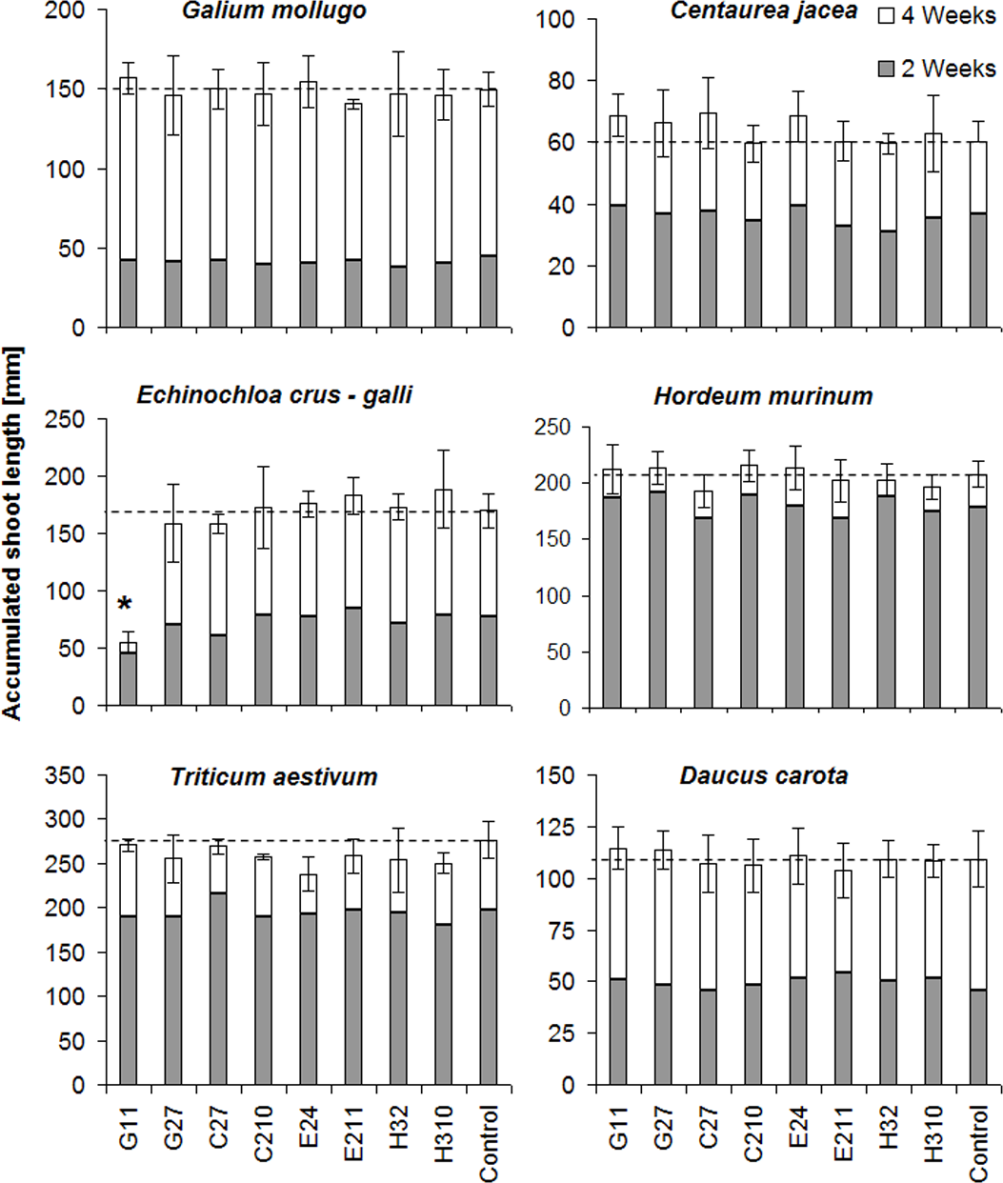
Influence of rhizobacteria inoculation on the shoot height of the model plants. Influence of eight rhizobacterial strains on the shoot height of four weed and two crop species after 2 and 4 weeks. The dotted line marks the average height of the plants in the control pots that did not receive rhizobacteria. The error bars indicate the standard deviation of the means (n = 4). * This bacterial strain x plant species combination differs significantly from all others (α = 0.001).

We found significantly differing aboveground biomass and shoot lengths between the six tested plant species (Table 2). Averaged across all eight bacterial strains and six plant species, the inoculation with rhizobacteria did not significantly affect the shoot height or the aboveground biomass of plants. However, the interaction term was highly significant (Table 2), indicating that particular plant species grew better or worse with particular bacterial strains (Fig. 2). We decomposed the interaction term into the contrasts “home clade” (bacterial strains from monocotyledonous plants on monocotyledonous plants and from dicotyledonous plants on dicotyledonous plants vs. other combinations) and “home species” (bacterial strains on their home vs. away plant species), but both of these were not significant. However, when we singled out the combination of bacterial strain G11 *(P. kilonensis/brassicacearum)*, a cyanide-producing strain isolated from *G. mollugo* roots, with the host species *E. crus-galli,* this contrast was highly significant (Table 2; Fig. 2). The aboveground biomass of the *E. crus-galli* plants with this bacterial strain was reduced by 95% compared with not-inoculated plants. This single-degree-of-freedom effect was so strong that it caused the above interaction term plant species x bacterial strain, with 40 degrees of freedom, to be significant.

**Table 2:** ANOVA-table for aboveground biomass in the host-plant selectivity experiment.

Plant Species	df 5	Mean sq 24597	F-value 16.37	P- value<0.0000
Bacterial strain	8	2625	1.75	0.0913
Contrasts within above term “Bacterial strain”:				
No bacteria vs. bacteria	1	2014	1.34	0.2487
Cyanide vs. non-cyanide producing bacteria	1	2155	1.43	0.2328
Residual of “Bacterial strain”^a^	6	2805	1.87	0.0895
**Plant species x bacterial strain**	**40**	**5400**	**3.59**	**< 0.0000**
Contrasts within above term “Plant species x bacterial strain”:				
*** Echinochloa*** ** x strain G11**	**1**	**153033**	**101.84**	**< 0.0000**
Residual of “Plant sp. x bacterial strain”^a^	39	1614	1.07	0.3680
Pot	192	1503	1.03	0.4023
*Residual*	*620*	*1462*		

Analysis of variance for aboveground biomass in the host-plant selectivity experiment. The indented lines show contrasts within the non-indented terms above them.

a These terms reflect the “difference” between the non-indented and the (sum of the) indented lines above them (see dfs). Significance level: α = 0.05.

Root samples were taken from the *E. crus-galli* pots without inoculation, with G11 inoculation or with G27 inoculation. A pot with G27 rhizobacteria was chosen as a model case in which our inoculation had no measurable effect on the growth of *E. crus-galli*. The roots of the *E. crus-galli* plants with G11 inoculation were extremely short while all others showed much stronger root development. MPN analysis revealed that more bacteria colonized roots in inoculations with G11 (1.6×10^9^ CFU g^−1^) than with G17 (1.7×10^8^ CFU g^−1^) or without inoculations (1.0×10^8^ CFU g^−1^). Because we only inoculated plants with fluorescent strains, the ratio of fluorescent to non-fluorescent bacteria was also recorded. We found twice as many fluorescent colonies in inoculations with G11 (18%) than with G27 (9%). No such colonies were found in the absence of inoculation. This indicates that G11 *(P. kilonensis/brassicacearum)* was more successful than other strains in colonizing the roots of *E. crus-galli*.

## Discussion

The identification of selected rhizobacteria allowed us to compare the microbial communities of four weedy plant species. We found that each plant species hosted a different set of soil microbes. Very few generalists which colonized the rhizosphere of more than one plant species were detected. Strains that were detected exclusively on the roots of one plant species were called specialists. The high ratio of specialists to generalists (25/2, Table 1) suggested that most rhizobacterial strains were adapted to only one of the four host plant species. Previous studies also reported high host-plant specificity of rhizosphere bacteria [20], [21]. Nevertheless, it is possible that some of our bacterial strains might have occurred in lower abundance on the other tested plant species and were therefore not detected with our method. Furthermore, we cannot rule out the possibility that they also occurred on other plant species not included in our experiment.

Our direct assessment of cyanide toxicity for the four weed and two additional crop species showed that these species differed in their ability to tolerate cyanide. In particular, three of the six species, *E. crus-galli*, *G. mollugo* and *D. carota*, were more sensitive and the three others, *T. aestivum*, *H. murinum* and *C. jacea*, were less sensitive to cyanide. This suggests that cyanide-producing rhizobacteria could negatively affect the growth of the first three species and thus potentially be used as selective herbicides. However, cyanide-tolerant plants could still be affected by other products of soil microbes such as phytotoxins or phytohormones.

In our main experiment we tested effects of single bacterial strains on the six plant species. As expected, we found significant growth differences between the six plant species. However, we could not relate plant aboveground biomass or shoot length to the treatment with bacterial strains that produced cyanide *in vitro*. It is conceivable that this was due to correlative beneficial effects of bacterial inoculation which may have compensated the negative effects. We hypothesised that cyanide-sensitive plant species would be more affected than cyanide-tolerant species. This was at least the case for the cyanide-sensitive species *E. crus-galli* which was affected negatively by the inoculation with one cyanide-producing bacterial strain. However, the absence of similar effects in the other two cyanide-sensitive species does not allow us to generalize.

Of all 48 plant species x bacterial strain combinations, only one strongly negative interaction, involving the exotic weed *E. crus-galli* and a cyanide-producing bacterial strain isolated from *G. mollugo,* was found. This suggests that single rhizobacterial strains, randomly assigned to plant species have only relatively small chances of causing negative growth effects. The single plant-bacterial inoculations used in the present study are an artificial setting that does not occur in nature. There are always large numbers of rhizobacterial species competing for a niche within the vicinity of roots. The finding of single species-pair effects suggest that it would be valuable to inoculate with mixtures of rhizobacteria to determine whether protective, synergistic or cumulative effects are observed in the plant. Increasing the complexity, however, would make it more difficult to allocate effects to a particular microorganism or interaction.

Analogous to observations in monoculture potato fields [22], we expected that plants would show stronger growth reductions when reared on soil with rhizobacteria isolated from roots of the same species (“home” bacteria) than with foreign (“away”) bacteria. By inoculating all plant species with the same bacterial isolates we could determine if home combinations affected plant growth more negatively than did away combinations. Our results do not support this “home/away” hypothesis. To the contrary, strain G11 *(P. kilonensis/brassicacearum)* isolated from *G. mollugo* roots dramatically reduced the growth of a different plant species, *E. crus-galli*. *Echinochloa crus-galli*, the only exotic among the six host plant species, does not share a common evolutionary history with this native weed species and its associated rhizobacteria.

It is possible that the contribution of microorganisms to the “home-away” effect found in soil from overused agricultural fields [22] is due to indirect interactions. DRBs were shown by others to suppress beneficial mycorrhizae or soil microbes [23]. Since we used sterile soil and seedlings, the inoculated rhizobacteria did not compete with other microbes or mycorrhiza. Therefore, we could not investigate such indirect effects. We suggest that experiments with microcosms of soil bacteria with or without mycorrhizae should be used to further investigate the “home vs. away” effect.

The host-selective effect of *P. kilonensis/brassicacearum* on *Echinochloa crus-galli* may have been caused by a combination of several factors [6]. First of all, the host plant must be recognized by the bacterium. For example, root exudates can create an environment that may favour strain-specific rhizobacterial colonizing [24]. Second, the bacteria must be able to occupy a niche in the rhizosphere of the host plant. In our experiments as well as in previous work investigating host-plant selectivity of DRBs, *Pseudomonas* strains were involved [6], [14], [25]. Strains of this genus have been shown to be highly competitive inhabitants of the rhizosphere and suitable to colonize and persist within this zone. [26]. Third, to cause host-selective reductions in plant growth, the bacterium (e.g. *P. kilonensis/brassicacearum)* must release effective compounds such as cyanide, phytotoxins or phytohormones which target susceptible plant species.

DRB that are weed-specific and do not harm crop plants could help to control invasive plant species or problem weeds in organic farming. We suggest that screening experiments to find host-plant selective rhizobacteria with herbicidal effects should take into account the factors listed above. The size of such experiments can be reduced by concentrating on cyanide-producing rhizobacteria if the target plant is sensitive to cyanide. The exclusive host-plant selectivity we described demonstrates the potential of rhizobacteria to act as taxonomically-targeted weed-control agents.
